# Engineering *Mycobacterium smegmatis* for testosterone production

**DOI:** 10.1111/1751-7915.12433

**Published:** 2016-11-17

**Authors:** Lorena Fernández‐Cabezón, Beatriz Galán, José L. García

**Affiliations:** ^1^ Department of Environmental BiologyCentro de Investigaciones BiológicasConsejo Superior de Investigaciones CientíficasRamiro de Maeztu 928040MadridSpain

## Abstract

A new biotechnological process for the production of testosterone (TS) has been developed to turn the model strain *Mycobacterium smegmatis* suitable for TS production to compete with the current chemical synthesis procedures. We have cloned and overexpressed two genes encoding microbial 17β‐hydroxysteroid: NADP 17‐oxidoreductase, from the bacterium *Comamonas testosteroni* and from the fungus *Cochliobolus lunatus*. The host strains were *M. smegmatis* wild type and a genetic engineered androst‐4‐ene‐3,17‐dione (AD) producing mutant. The performances of the four recombinant bacterial strains have been tested both in growing and resting‐cell conditions using natural sterols and AD as substrates respectively. These strains were able to produce TS from sterols or AD with high yields. This work represents a proof of concept of the possibilities that offers this model bacterium for the production of pharmaceutical steroids using metabolic engineering approaches.

## Introduction

**Figure 1:**
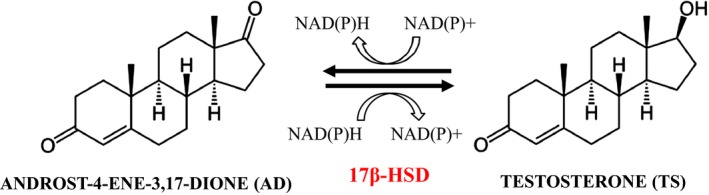
Schematic representation of transformation process of AD into TS by 17β‐HSD.

Testosterone (TS) is one of the oldest drugs used in medicine and has a long efficacy and safety record for hormone replacement therapy in men with androgen deficiency. Currently, TS is chemically produced from androst‐4‐ene‐3,17‐dione (AD) (Ercoli and Ruggierii, 1953). In mammals, the synthesis of TS from AD is catalysed by the microsomal 17‐ketosteroid reductase (17β‐HSD; 17β‐hydroxysteroid:NADP 17‐oxidoreductase, EC 1.1.1.64) (Bogovich and Payne, 1980) (Fig. 1). Up to now, 14 different subtypes of 17β‐HSD have been identified in mammals and most of them belong to the short‐chain dehydrogenase:reductase superfamily (SDR). They catalyse NAD(P)H/NAD(P)^+^‐dependent reductions/oxidations at the C‐17 position of different steroids (Peltoketo *et al*., 1999; Moeller and Adamski, 2006, 2009; Marchais‐Oberwinkler *et al*., 2011). The majority of 17β‐HSD enzymes are able to catalyse, at least to some extent, reverse reactions under *in vitro* conditions. In the presence of a substantial excess of a suitable cofactor and/or in the absence of the preferred cofactor, 17β‐HSD can be compelled to catalyse both oxidative and reductive reactions. Based on this property, a process has been developed to produce *in vitro* TS from AD using the recombinant murine 17β‐HSD type V (aldo‐keto‐reductase instead of SDR family) and glucose dehydrogenase as cofactor recycling enzyme (Fogal *et al*., 2013). However, due to the high cost of the process, it is not currently used for industrial purposes.

On the other hand, enzymatic reduction of AD to TS by 17β‐HSD has also been described in different microorganisms (Donova *et al*., 2005), including bacteria (Schultz *et al*., 1977; Payne and Talalay, 1985; Sarmah *et al*., 1989; Liu *et al*., 1994; Egorova *et al*., 2002a,b, 2005), yeasts (Ward and Young, 1990; Singer *et al*., 1991; Długoński and Wilmańska, 1998; Pajic *et al*., 1999), filamentous fungi (Kristan and Rižner, 2012) and plants (Hamada and Kawabe, 1991). Moreover, a single‐step microbial transformation process has been reported for the production of TS from sterols using several *Mycobacterium* sp. mutants (Wang *et al*., 1982; Hung *et al*., 1994; Liu *et al*., 1994; Llanes *et al*., 1995; Liu and Lo, 1997; Borrego *et al*., 2000; Lo *et al*., 2002; Mei *et al*., 2005; Egorova *et al*., 2009). During the course of this work, Dlugovitzky *et al*. (2015) have shown that *Mycobacterium smegmatis* PTCC 1307 was able to produce TS and other estrogens from tritiated precursors. However, TS has not been detected as a metabolic intermediate when *M. smegmatis* mc^2^155 is cultured in the presence of phytosterols or cholesterol, neither in the wild‐type strain nor in the AD‐producing strain (Galán *et al*., unpublished), unlike in other mycobacterial species (Wang *et al*., 1982; Smith *et al*., 1993; Egorova *et al.,* 2002b). This observation suggests that *M. smegmatis* mc^2^155 does not contain a functional gene encoding a 17β‐HSD or at least, it is not induced in the presence of these compounds.

Although several microbial 17β‐HSD enzymes have been cloned and characterized (Abalain *et al*., 1993; Rižner *et al*., 1999; Chang *et al*., 2010), none of them were used to develop genetically engineered bacteria to improve the biotechnological production of TS. These genes have been only expressed in *Escherichia coli*, a bacterium unable to efficiently transport sterols or AD, impairing the development of an industrial biotransformation processes.

**Figure 2:**
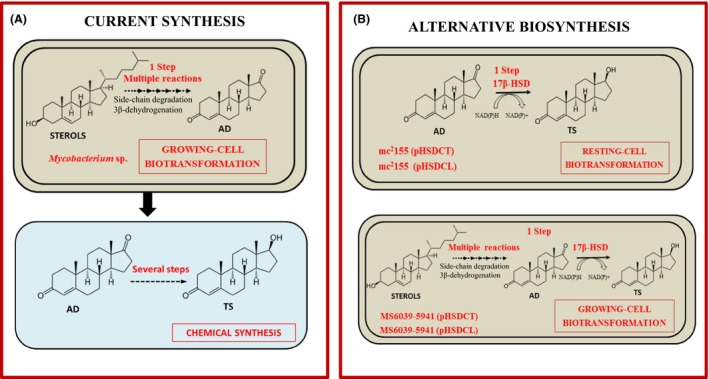
Methods for TS synthesis. (A) Current synthesis of TS at the pharmaceutical industry. First, biotransformation process for the production of AD from sterols is carried out by *Mycobacterium* sp. Second, AD is transformed into TS by a chemical process. (B) Alternative production of TS proposed in this work by recombinant *M. smegmatis* strains overexpressing 17β‐HSD‐encoding genes. The biotransformation of AD into TS can be achieved by resting‐cell in the strains *M. smegmatis* mc^2^155 (pHSDCT) and *M. smegmatis* mc^2^155 (pHSDCL). The production of TS from sterols can be realized by growing‐cell biotransformations in the mutant strains *M. smegmatis* MS60369‐5941 (pHSDCT) and *M. smegmatis* MS60369‐5941 (pHSDCL).

The aim of this work was to develop recombinant bacteria overexpressing 17β‐HSD genes that will be able to efficiently biotransform either natural sterols (e.g. phytosterols or cholesterol) or AD into TS in order to compete with the current chemical synthesis of TS by using a new biotechnological process. To fulfil this goal, we have cloned and overexpressed the genes encoding two microbial 17β‐HSDs, from the bacterium *Comamonas testosteroni* (Abalain *et al*., 1993) and from the fungus *Cochliobolus lunatus* (Rižner *et al*., 1999), using as hosts the wild‐type *M. smegmatis* and an AD‐producing mutant of this bacterium. The performances of the new created recombinant bacterial strains have been tested both in growing and resting‐cell conditions using sterols and AD as substrates respectively (Fig. 2).

## Results and discussion

### Working hypothesis and selection of 17β‐HSD encoding genes

Up to now, to our knowledge, there is not an example of any engineered bacterium able to produce TS from sterols or AD. In this sense, we decided to investigate if *M. smegmatis* could be a suitable chassis for this purpose. The selection of *M. smegmatis* to achieve TS production is mainly based in two properties: first, it is not able to degrade AD and second, there are evidences that AD can be efficiently transported (L. Fernández‐Cabezón *et al.,* unpublished). Therefore, the circumvention of the bacterial mineralization of AD and TS during the biotransformation process is not a requirement. We have already evidenced that this fast‐growing and non‐pathogenic bacterium, which is able to transport and metabolize cholesterol and phytosterols, can be a suitable cell factory for the industrial production of steroid intermediates such as AD using sterols as feedstock (Galán *et al.,* unpublished). Other steroid‐metabolizing bacteria that are able to transport AD (e.g. *Comamonas, Rhodococcus* or *Gordonia*) cannot be in principle used as an alternative host because they degrade AD and TS efficiently (Tamaoka *et al*., 1987; Cabrera *et al*., 2000; Fernández de las Heras *et al*., 2009; Li *et al*., 2014). Taking into account that we were not able to identify in *M. smegmatis* mc^2^155, a functional gene encoding a 17β‐HSD, the aim of this work was to overproduce a 17β‐HSD obtained from a heterologous organism either in the wild‐type or the AD‐producing mutant strains. In this way, the *M. smegmatis* recombinant strains can be utilized to transform AD into TS by a resting‐cell system or to produce TS from sterols by a fermentation process (Fig. 2).

As the genes encoding 17β‐HSD enzymes from mycobacterial species have not been identified and these proteins have been only partially purified and characterized (Goren *et al*., 1983; Egorova *et al*., 2002a, 2005), we initially selected as enzyme candidates for metabolic engineering the well‐described 17β‐HSDs from the bacterium *C. testosteroni* (Schultz *et al*., 1977; Lefebvre *et al*.,^1979^; Minard *et al*., 1985 *;* Genti‐Raimondi *et al*., 1991; Yin *et al*., 1991; Abalain *et al*., 1993; Benach *et al*., 1996, 2002; Oppermann *et al*., 1997; Cabrera *et al*., 2000) and the fungus *C. lunatus* (Plemenitas *et al*., 1988; Rižner *et al*., 1996, 1999, 2000, 2001a,b; Rižner and Zakelj‐Mavric, 2000; Zorko *et al*., 2000; Kristan *et al*., 2003, 2005, 2007a,b; Cassetta *et al*., 2005; Ulrih and Lanisnik Rižner, 2006; Brunskole *et al*., 2009; Svegelj *et al*., 2012), because both enzymes present some relevant differences. Although they catalyse a reversible reaction and display similar reaction mechanisms, the reaction equilibrium of the fungal 17β‐HSD is shifted towards reduction, whereas the bacterial enzyme is shifted towards oxidation, as this enzyme is mainly involved into the TS catabolism in *C. testosteroni* (Genti‐Raimondi *et al.,* 1990; Cabrera *et al*., 2000). Moreover, the fungal 17β‐HSD prefers NADPH as coenzyme for reduction of AD, while preferences between NAD^+^/NADP^+^ are not observed for oxidation of TS (Rižner and Zakelj‐Mavric, 2000). The bacterial enzyme is in fact a 3β/17β‐HSD, this is a bifunctional enzyme with a single catalytic site able to accommodate both the 3β‐ and 17β‐activities, and uses NAD(H) as cofactor (Minard *et al*., 1985; Benach *et al*., 2002). These issues are relevant for developing a biotechnological process as the cellular content of NAD(H) and/or NADP(H) will determine the direction of the catalytic reaction and the yield of TS production. Remarkably, Xu *et al*. (2016) have described the Hsd4A protein of *M. neoaurum* ATCC 25795 as a dual‐function enzyme, with both 17β‐HSD and β‐hydroxyacyl‐CoA dehydrogenase activities *in vitro*. However, the 17β‐HSD enzyme does not appear to be reversible *in vitro* as it is able to transform TS into AD but not AD into TS (Xu *et al*., 2016).

As an alternative to the 17β‐HSD enzymes of *C. testosteroni* and *C. lunatus,* we have tried to identify homologous enzymes in other microorganisms. For instance, we found mycobacterial proteins with a small identity (<40%) to the 17β‐HSD enzyme from *C. testoteroni*. In particular, several putative homologue short‐chain dehydrogenases were found in *M. smegmatis* mc^2^155, such as the 3‐α‐(or 20‐β)‐hydroxysteroid dehydrogenase (*MSMEG_3515*, 38% identity), and the cyclopentanol dehydrogenase (*MSMEG_6709*, 37% identity). Analysing the genomic context of such genes, non‐relationship with steroid degradative enzymes was found. On the other hand, the 17β‐HSD from *C. lunatus* presents a high sequence identity (60–95%) with proteins from the fungal *Leotiomyceta* group, which belongs to Ascomycota Phylum. These identities are not present in other representatives of this phylum, such as the *Saccharomyces* genus. However, 17β‐HSD activity was detected in *Saccharomyces cerevisiae* and other fungi (Ward and Young, 1990; Długoński and Wilmańska, 1998). Outside *Leotiomyceta* group, identities of 42–48% are found in bacteria of different phyla (Cyanobacteria, different groups of *Proteobacteria*,* Firmicutes*, etc.), including members of actinobacteria (e.g. *Mycobacterium abscessus*,* Rhodococcus wratislaviensis* NBRC 100605 or several *Streptomyces* species). In *M. smegmatis*, only proteins with an identity lower than 36% are found. Consequently, the correlation between protein identity and 17 β‐HSD activity, as well as their true biological role in the native organisms, cannot be easily established by *in silico* analysis. In fact, this observation has been also described in vertebrates whose 17 β‐HSD enzymes show generally low sequence similarity (15–20%) (Moeller and Adamski, 2009). Therefore, on the light of these analyses, finally the 17β‐HSD enzymes from *C. testosteroni* and *C. lunatus* were selected as the best choice to carry out our work.

### Engineering heterologous 17β‐HSDs in recombinant *M. smegmatis* strain

We have cloned the genes encoding the 17 β‐HSDs from *C. testosteroni* and *C. lunatus* in pMV261, an *E. coli*/*M. smegmatis* shuttle vector, under the control of the constitutive *Phsp60* promoter, rendering the plasmids pHSDCT and pHSDCL respectively (see Experimental procedures). Both plasmids were transformed in both, the *M. smegmatis* wild‐type strain and the AD‐producing *M. smegmatis* MS6039‐5941 mutant, generating four recombinant strains, i.e. *M. smegmatis* mc^2^155 (pHSDCT), *M. smegmatis* mc^2^155 (pHSDCL), *M. smegmatis* MS6039‐5941 (pHSDCT) and *M. smegmatis* MS6039‐5941 (pHSDCL) (Table 1).

**Table 1:** List of bacterial strains, plasmids and primers used in this study

Strains or plasmids	Genotype and/or description	Source or reference
Strains		
*Mycobacterium "smegmatis*		
mc^2^155	*ept‐1*, mc²6 mutant efficient for electroporation	Snapper *et al*. (1990)
MS6039‐5941	mc^2^155 mutant Δ*MSMEG_6039* Δ*MSMEG_5941*	Galán *et al*. (unpublished)
mc^2^155 (pHSDCT)	mc^2^155 harbouring plasmid pHSDCT	This study
mc^2^155 (pHSDCL)	mc^2^155 harbouring plasmid pHSDCL	This study
MS6039‐5941(pHSDCT)	MS6039‐5941 harbouring plasmid pHSDCT	This study
MS6039‐5941(pHSDCL)	MS6039‐5941 harbouring plasmid pHSDCL	This study
*Escherichia coli*		This study
DH10B	F^*−*^ *, mcrA*, Δ (*mrrhsdRMS‐mcrBC*), Φ80d*lacZ*ΔM15, Δ*lacX74*,* deoR, recA1, araD139,* Δ(*ara‐leu*)7697, *galU, galK*, λ^−^, *rpsL, endA1, nupG*	Invitrogen
DH10B (pUC57‐17HSD)	DH10B strain harbouring plasmid pUC57‐17HSD	This study
DH10B (pGEMT‐HSDCT)	DH10B strain harbouring plasmid pGEMT‐HSDCT	This study
DH10B (pHSDCT).	DH10B strain harbouring plasmid pHSDCT	This study
*Comamonas testosteroni*		
ATCC^®^ 11996^™^		ATCC
*Plasmids*		
pMV261	*Mycobacterium*/*E. coli* shuttle vector with the kanamycin resistance *aph* gene from transposon Tn903 and the promoter from the *hsp6*0 gene from *M. tuberculosis*	Stover *et al*. (1991)
pGEM^®^‐T Easy	*E. coli* cloning vector; Amp^R^; T7 and SP6 RNA polymerase promoters flanking a multiple cloning region within the α‐peptide coding region of β‐galactosidase for the identification of recombinants by blue/white screening	Promega
pGEMT‐HSDCT	pGEMT‐Easy harbouring the gene encoding the 17β‐HSD from *C. testosteroni*	This study
pUC57‐17HSD	pUC57 harbouring the synthetic gene encoding the 17β‐HSD from *C. lunatus*	This study
pHSDCT	pMV261 harbouring the gene encoding the 17β‐HSD from *C. testosteroni*	This study
pHSDCL	pMV261 harbouring the synthetic gene encoding the 17β‐HSD from *C. lunatus*	This study
*Primers*		
HDHF	AGAGGAGATATACCATGGGCAGCAGCCATCATCATCATCATCACACAAATCGTTTGCAGGGTAAGG	This study
HDHR	AAGCTTCTATAGCCCCATGCCCAGAATCG	This study

### Production of TS from AD by resting‐cell biotransformation

**Figure 3:**
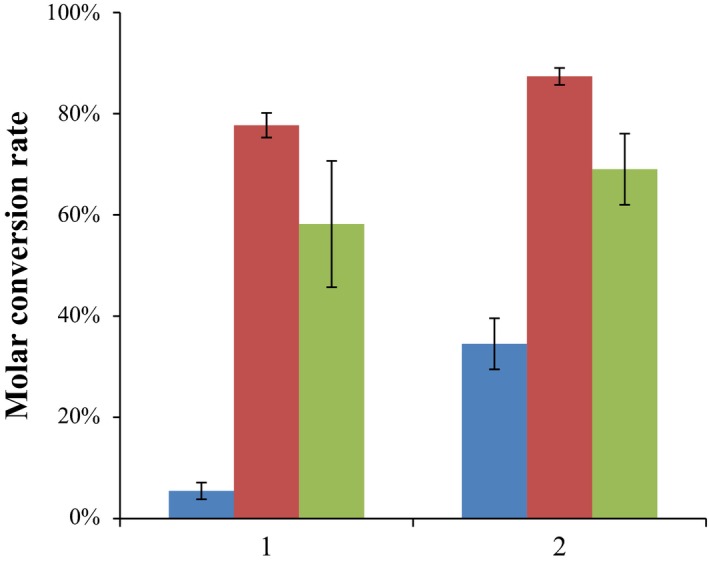
Production of TS by resting‐cell processes. The conversion of AD by the strains *Mycobacterium smegmatis* mc^2^155 (pHSDCT) (1) and *M. smegmatis* mc^2^155 (pHSDCL) (2) was tested in three culture conditions: no carbon source addition (blue), 1% glucose (red) and 1% glycerol (green). Average and standard deviation of two biological replicates at 24 h of culture are represented.

The ability of the *M. smegmatis* (pHSDCT) and *M. smegmatis* (pHSDCL) recombinant strains to produce TS from AD was tested using a resting‐cell assay. In the standard conditions (no additional carbon sources present in the reaction medium), small amount of TS was detected (Fig. 3). However, when the reaction medium was supplemented with 1% glucose, the substrate AD was efficiently transformed into TS using both strains (Fig. 3). The biotransformation was slightly more efficient using the recombinant bacteria harbouring the fungal gene [i.e. *M. smegmatis* (pHSDCL)]. When the reaction medium was supplemented with 1% glycerol instead of glucose, the biotransformation was slightly less efficient (Fig. 3). This result suggest that the intracellular NAD(P)^+^/NAD(P)H ratio could be different in the presence of glucose or glycerol being critical for the process. In this sense, the effect of a carbon source supplementation and also the pH have been already demonstrated to be determinant in the reaction equilibrium for the TS production (Liu *et al*., 1994; Llanes *et al*., 1995; Liu and Lo, 1997; Egorova *et al*., 2009).

The use of the recombinant *M. smegmatis* MS6039‐5941 (pHSDCT) and *M. smegmatis* MS6039‐5941 (pHSDCL) strains instead the wild‐type recombinants does not provide any significant advantage in the resting‐cell process because, as mentioned above, both the wild‐type and MS6039‐5941 mutant strains are unable to metabolize AD or TS.

### Production of TS from sterols by growing‐cells biotransformation

**Figure 4:**
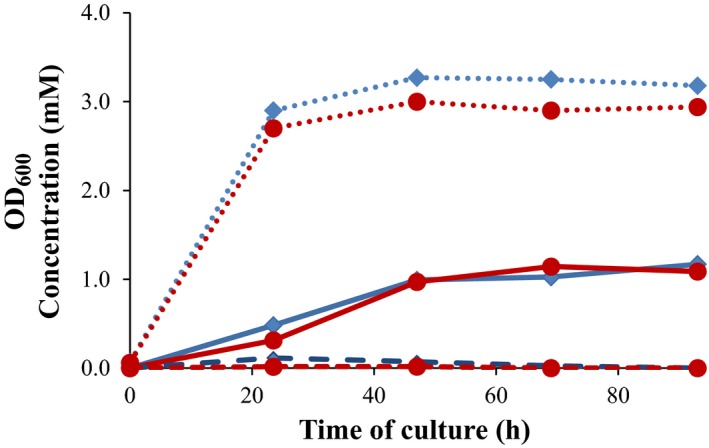
Production of TS from cholesterol by growing‐cell processes. The strains *Mycobacterium smegmatis* MS6039‐5941 (pHSDCT) (red) and *M. smegmatis* MS6039‐5941 (pHSDCL) (blue) were growth at minimal medium containing 18 mM glycerol (carbon and energy source) and 1.8 mM cholesterol (substrate). The AD concentration (continuous lines) and TS (dashed lines) and DO_600_ (dotted line) are represented. A representative experiment is shown.

The ability to produce TS from natural sterols in a single biotransformation step was tested by growing *M. smegmatis* MS6039‐5941 (pHSDCT) and *M. smegmatis* MS6039‐5941 (pHSDCL) strains in minimal medium containing 18 mM glycerol as a main carbon and energy source and 1.8 mM cholesterol as a substrate. In this culture condition, the two recombinant strains transformed the cholesterol into AD but TS was only detected in small traces during the exponential growth phase (Fig. 4). Based on the resting‐cell results, we tested the production of TS from sterols using 9 mM glucose instead of 18 mM glycerol as a carbon source obtaining similar results those using glycerol (data not shown). It is worth to mention that, in this culture condition, cell growth is only observed during the first 24 h due to the complete consumption of glycerol or glucose. After this time, the cholesterol is still transformed into AD but the cholesterol side‐chain degradation might not supply enough carbon and/or energy to support cell division (Fig. 4). Moreover, the reduction of AD into TS is not efficient in nutrient‐limited conditions and an alternative carbon source could be necessary for reactivating cell metabolism.

**Figure 5:**
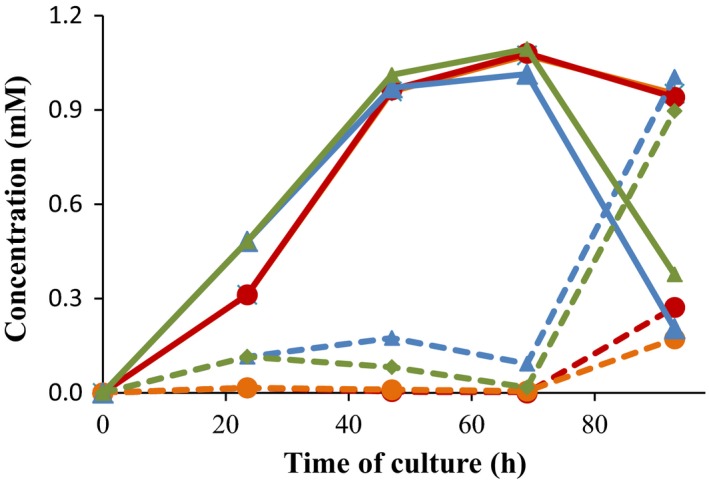
Production of TS from cholesterol by a pseudo‐resting‐cell process. The strains *Mycobacterium smegmatis* MS6039‐5941 (pHSDCT) and *M. smegmatis* MS6039‐5941 (pHSDCL) were growth at minimal medium containing 18 mM glycerol (carbon and energy source) and 1.8 mM cholesterol (substrate) and, 1% glucose or glycerol was added at 69 h of culture. The following cultures were tested: MS6039‐5941 (pHSDCT) with glucose (red); MS6039‐5941 (pHSDCT) with glycerol (orange); MS6039‐5941 (pHSDCL) with glucose (blue) and MS6039‐5941 (pHSDCL) with glycerol (green). The AD concentration (continuous lines) and TS (dashed lines) are represented. A representative experiment is shown.

As the biotransformation of AD into TS appears to be dependent of the NAD(P)/NAD(P)H cofactor balance, we simulated a pseudo‐resting‐cell system carrying out two consecutive biotransformation steps at the same shake flask. The first step was carried out in the presence of 18 mM glycerol and 1.8 mM cholesterol. In these conditions, we have observed that the cholesterol is mostly depleted and transformed into AD before 69 h of culture (data not shown). So at this moment, in a second step, we added glucose or glycerol and measured the production of TS after 24 h as it was done for the resting‐cell assays. Using this approach, the two recombinant strains were able to transform sterols into TS more efficiently, although significant differences were observed probably due to kinetic differences between both 17β‐HSD enzymes (Fig. 5). In the presence of 1% glucose, the average rate of TS to androstenes (AD and TS) was 77.6% and 28.6% using the strains MS6039‐5941 (pHSDCL) and MS6039‐5941 (pHSDCT) respectively. Similar results were obtained when glucose was used as an initial carbon source and glucose was added at 69 h of culture (data no shown). However, this average rate was slightly lower when an addition of glycerol instead of glucose was supplied to both strains (Fig. 5).

**Figure 6:**
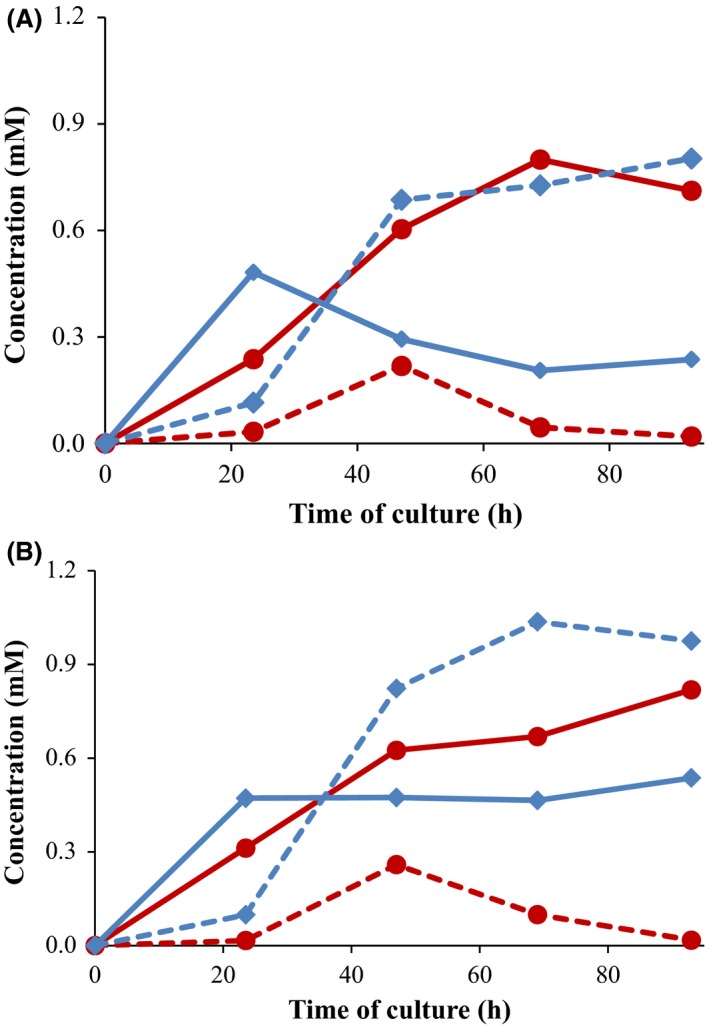
Production of TS from cholesterol by growing‐cell processes. The *Mycobacterium smegmatis* MS6039‐5941 (pHSDCT) (red) and *M. smegmatis* MS6039‐5941 (pHSDCL) (blue) strains were grown in minimal medium containing 1.8 mM cholesterol (substrate) and an alternative carbon source: (A) 18 mM glycerol with an addition of 1% glucose at 24 h; (B) 1% glucose without any addition The AD concentration (continuous lines) and TS (dashed lines) are represented. A representative experiment is shown

**Figure 7:**
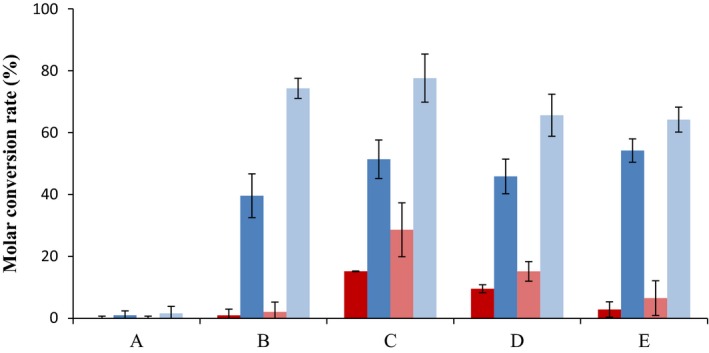
Conversion rates of TS in growing‐cell processes. The strains *Mycobacterium smegmatis* MS6039‐5941 (pHSDCT) (red bars) and *M. smegmatis* MS6039‐5941 (pHSDCL) (blue bars) were grown at minimal medium containing 1.8 mM cholesterol (substrate) and 18 mM glycerol (carbon and energy source). The following culture conditions were tested: (A) Without addition of any carbon source; (B) with addition of 1% glucose at 24 h; (C) with addition of 1% glucose at 69 h; (D) with addition of 1% glycerol at 69 h; (E) without addition and 1% glucose instead of glycerol as initial substrate. The molar conversion rate of TS (first and second bars) and the ratio of TS to androstenes (third and fourth bars) at the end of the culture (93 h) are shown. The molar conversion rate was calculated on the basis of 1.8 mM cholesterol added. Androstenes are calculated by adding AD and TS. Average and standard deviation of two biological replicates are represented.

To test the influence of bacterial metabolic state on the TS production, other culture conditions were assayed. First, we tested the addition of glucose at the late‐exponential growth phase (e.g. 23.5 h) (Fig. 6). Second, we added higher concentrations of carbon source at the beginning of the culture (e.g. 55.5 mM glucose (1% glucose) instead of 18 mM glycerol) (Fig. 6). In both culture conditions, the recombinant strains were able to produce TS from cholesterol but their behaviour was different. The strain MS6039‐5941 (pHSDCL) produced higher TS yields and the reversion of TS to AD was not significant. However, the strain MS6039‐5941 (pHSDCT) produced TS but in this case a notable reconversion of TS into AD was observed after 47 h of culture. The molar conversion rates and the TS to androstenes ratios are shown in Fig. 7. According to these results, the production of TS from sterols is achieved more efficiently with the strain MS6039‐5941 (pHSDCL) than with the strain MS6039‐5941 (pHSDCT). Although the presence of an additional carbon source (i.e. glycerol or glucose) is required for the production of TS for both recombinant strains, the reconversion of TS into AD is not observed in the case of the fungal 17β‐HSD‐expressing strain once the carbon source has been consumed. This reversibility was described previously in other mycobacterial mutants producing TS from cholesterol (Liu and Lo, 1997). These results reinforce the use of the fungal enzyme as the best candidate to genetically engineer the recombinant strains to produce TS from sterols.

### Concluding remarks

We have demonstrated that *M. smegmatis* is an excellent chassis to develop biotechnological processes for the biotransformation of sterols and their derivatives into valuable pharmaceutical compounds. The current genetic tools available to transform this organism have allowed for expressing stably two genes coding 17β‐HSDs enzymes. Our recombinant strains were able to produce TS from AD and/or from sterols with high yields that are comparable with previously published data using mycobacterial strains obtained by conventional mutation procedures (Liu *et al*., 1994; Liu and Lo, 1997). Moreover, our rational approach makes possible the introduction of heterologous 17β‐HSD enzymes from diverse origins with different catalytic properties (e.g., substrate and cofactor specificity) offering more versatility than conventional methods. Some of the 17β‐HSD activities described in the conventional mutants appears to have different catalytic properties. In fact, in some mycobacterial strains, the double reduction of ADD [both of 17‐keto group and 1(2)‐double bound] was more effective for TS formation than a single reduction of 17‐keto group of AD (Hung *et al*., 1994; Egorova *et al*., 2009).

This work represents a proof of concept of the possibilities of using this model bacteria and metabolic engineering approaches for the production of pharmaceutical steroids. However, additional efforts are needed to optimize the production of TS from sterols in a single biotransformation step. Two factors appear to be determinant for the improvement of this process: the reversibility of 17β‐HSD enzymes and the cell metabolic state. To overcome the first of these factors, works to identify non‐reversible 17β‐HSDs and/or to modify the characterized enzymes by protein engineering can be explored. Several *in vitro* attempts have been already done to design rationally 17β‐HSD mutants from *C. testosteroni* and *C. lunatus* which present alterations in substrate specificity and/or coenzyme requirements, as well as improvements overall catalytic activity (Oppermann *et al*., 1997; Kristan *et al*., 2003, 2005, 2007a,b; Brunskole *et al*., 2009; Svegelj *et al*., 2012). To overcome the second factor, the control of bacterial metabolism by metabolic engineering and systems biology approaches would be useful. Factors such as the supplementation of carbon source, pH or mode of substrate addition, have been proved to be determinant for the reduction of AD to TS. Systems biology approaches could give more detailed information about microbial metabolism and how to increase TS yields.

## Experimental procedures

### Chemicals

4‐Androstene‐3,17‐dione (AD) was purchased from TCI America. Chloroform, glucose and glycerol were purchased from Merck (Darmstardt, Germany). Methanol and acetonitrile of HPLC quality were purchased from Scharlau (Sentmenat, Spain). Cholesterol, TS, Tween 80, tyloxapol, ampicilin and kanamycin were from Sigma (Steinheim, Germany).

### Bacterial strains, plasmids and culture conditions

The bacterial strains, plasmids and primers used in this work are listed in Table 1. *Mycobacterium smegmatis* strains were grown at 37°C in an orbital shaker at 200 rpm. Middlebrook 7H9 broth medium (Difco) supplemented with 0.4% glycerol and 0.05% Tween 80 was used as rich medium. 7H9 broth without any supplement was used as minimal medium. 7H10 agar (Difco) plates supplemented with 10% albumin‐dextrose‐catalase (Becton Dickinson) were used for solid media. Kanamycin (20 μg ml^−1^) was used for strain selection when appropriate.

*Escherichia coli* DH10B strain was used as host for cloning purposes. It was grown in LB medium at 37°C in an orbital shaker at 200 rpm. LB agar plates were also used for solid media. Ampicilin (100 μg ml^−1^) or kanamycin (50 μg ml^−1^) were used for plasmid selection and maintenance.

*Comamonas testosteroni* ATCC 11996 was grown in LB medium at 30°C in an orbital shaker at 200 rpm to extract genomic DNA.

### DNA manipulations and sequencing

DNA manipulations and other molecular biology techniques were essentially as described by Sambrook and Russell (2001). Isolation of *C. testosteroni* genomic DNA was performed with the Bacteria Genomic Prep Mini Spin Kit (GE Healthcare). Oligonucleotides were purchased from Sigma‐Aldrich. DNA amplification was performed on a Mastercycler Gradient (Eppendorf) using DNA polymerase I and Pfu polymerase from Biotools B. M. Labs. Reaction mixtures contained 1.5 mM MgCl_2_ and 0.25 mM dNTPs. DNA fragments were purified with High Pure PCR System Product Purification Kit (Roche). Restriction enzymes were obtained from various suppliers and were used according to their specifications. Plasmid DNA was prepared with a High Pure Plasmid Isolation Kit (Roche Applied Science). *Escherichia coli* was transformed by the rubidium chloride method (Wirth *et al*., 1989). *Mycobacterium smegmatis* cells were transformed by electroporation (Gene Pulser; Bio‐Rad) (Parish and Stoker, 1998). All cloned inserts and DNA fragments were confirmed by DNA sequencing through an ABI Prism 377 automated DNA sequencer (Applied Biosystems Inc.) at Secugen S.L. (Madrid, Spain).

### Construction of plasmids pHSDCT and pHSDCL

To isolate the gene encoding the 3β/17β‐HSD from *C. testosteroni* ATCC 11996*,* the genomic DNA was isolated and amplified the gene by PCR using the oligonucleotides HDHF and HDHR (Table 1). A sequence coding six histidines was inserted in the oligonucleotide HDHF, generating a novel gene version encoding a modified 17β‐HSD from *C. testosteroni* that contains a polyhistidine‐tag inserted in the N‐terminal end. The amplified 812 bp fragment was cloned into pGEM^®^‐T Easy (Promega) to generate the plasmid pGEMT‐HSDCT using *E. coli* DH10B as host. The cloned fragment was further subcloned into the plasmid pMV261 able to replicate in *E. coli* and *Mycobacterium*. For this goal, the plasmid pGEMT‐HSDCT was digested with EcoRI and HindIII and the fragment was ligated with the vector pMV261 cut with the same restriction enzymes generating the plasmid pHSDCT. This plasmid was transformed into *E. coli* DH10B to generate the recombinant strain *E. coli* DH10B (pHSDCT). The plasmid pHSDCT isolated from *E. coli* was transformed by electroporation into *M. smegmatis* mc^2^155 and the AD‐producing mutant *M. smegmatis* MS6039‐5941 competent cells, generating the recombinant strains *M. smegmatis* mc^2^‐155 (pHSDCT) and *M. smegmatis* MS6039‐5941 (pHSDCT) (Table 1).

**Figure 8:**
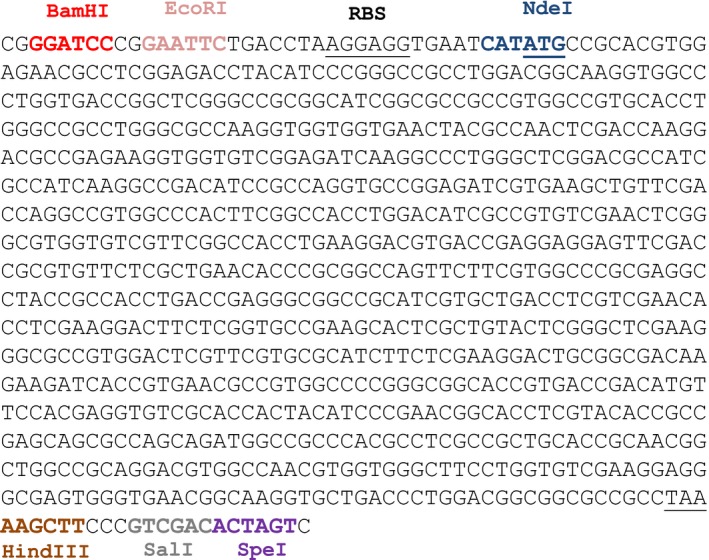
Gene sequence encoding the 17β‐HSD from *C. lunatus* chemically synthesized with an optimized codon usage for its expression in *Mycobacterium*. The ribosome‐binding site (RBS), start codon and stop codon are underlined. Several recognition sites by restriction enzymes were incorporated to facilitate subcloning tasks.

The gene encoding the 17β‐HSD from *C. lunatus* was chemically synthesized with an optimized codon usage for its expression in *Mycobacterium* (ATG:biosynthetics GmbH, Germany) (Fig. 8). The codon optimization was carried out using the program OPTIMIZER (Puigbò *et al*., 2007) and the codon usage table found in KDRI (Kazusa DNA Research Institute, Japan). The synthetic gene was supplied cloned into the EcoRV site of the commercial vector pUC57 generating the plasmid pUC57‐17HSD. This plasmid was transformed into *E. coli* DH10B generating the recombinant strain *E. coli* DH10B (pUC57‐17HSD). The fragment containing the synthetic gene encoding the 17β‐HSD was subcloned into the plasmid pMV261. For this purpose, the pUC57‐17HSD plasmid was digested with EcoRI and HindIII and the fragment was ligated to the pMV261 vector digested with the same restriction enzymes. Thus, we created the plasmid pHSDCL which was transformed into *E. coli* DH10B to generate the recombinant *E. coli* DH10B (pHSDCL). The plasmid pHSDCL extracted from *E. coli* was transformed by electroporation into *M. smegmatis* mc^2^155 and *M. smegmatis* MS6039‐5941 competent cells to generate the recombinant strains *M. smegmatis* mc^2^155 (pHSDCL) and *M. smegmatis* MS6039‐5941 (pHSDCL) respectively.

### Resting‐cell biotransformations

The recombinant strains were grown in rich medium at 37°C during 24 h. The cells were harvested by centrifugation at 5000×***g*** for 20 min at 4°C and washed once with 0.85% NaCl. The biotransformation was carried out with an optical density (OD_600_) of 18 in a 100 ml shake flask containing 40 ml of reaction mixture: 0.1 M phosphate buffer (pH 8.0), 2 mM AD (substrate) and 0.05% Tween 80. An additional carbon source was added in some cases. AD was incorporated into the medium as a solution with randomly methylated β‐cyclodextrin (1:10.3, molar ratio) (Klein *et al*., 1995).

### Growing‐cell biotransformations

The precultures of the recombinant strains were grown in rich medium at 37°C during 24 h and used to inoculate 30 ml of 7H9 minimal medium containing 1.8 mM cholesterol (substrate), 18 mM glycerol (carbon and energy source), 3.6% tyloxapol and 20 μg ml^−1^ kanamycin. Different concentrations of glucose instead of glycerol were also tested. The cholesterol was dissolved in 10% tyloxapol prior to its addition to the minimal medium. Due to the low solubility of this steroid, a stock solution was warmed at 80°C in agitation, sonicated in a bath for 1 h and then autoclaved. The cultures were grown in 100 ml shake flasks at 37°C in an orbital shaker at 200 rpm. An addition of 1% glucose or 1% glycerol was added at different times of culture.

### Analytical methods

The culture broth was extracted with chloroform (0.5:1, v/v) twice. The organic phase was evaporated and the residue was dissolved in acetonitrile. AD and TS were determined by reversed‐phase HPLC using a Teknokroma mediterranea^™^ Sea_18_ column (15 cm × 0.46 cm; 5 μM) and UV detection at 240 nm. Mobile phase was composed of methanol and water (75/25, v/v), flow rate 0.85 ml min^−1^. AD and TS were used as standards. The conversion rate of TS was calculated on the basis of cholesterol added to the medium (growing‐cell biotransformations) or AD measured into the sample (resting‐cell biotransformations).

### Bioinformatic analysis

Sequence alignments were carried out using clustal w (Thompson *et al*., 1994) and different BLAST algorithms from the National Centre of Biotechnology Information Server (NCBI) were also used.

## Conflict of interest

None declared.
